# Data Mining in the Development of Mobile Health Apps: Assessing In-App Navigation Through Markov Chain Analysis

**DOI:** 10.2196/11934

**Published:** 2019-06-07

**Authors:** Jeroen Stragier, Gilles Vandewiele, Paulien Coppens, Femke Ongenae, Wendy Van den Broeck, Filip De Turck, Lieven De Marez

**Affiliations:** 1 imec-mict Department of Communication Sciences Ghent University Ghent Belgium; 2 imec-IDLab Department of Information Technology Ghent University Ghent Belgium; 3 imec-smit Department of Communication Sciences Vrije Universiteit Brussel Brussels Belgium

**Keywords:** eHealth, mHealth, Markov Chain, log data, data analytics

## Abstract

**Background:**

Mobile apps generate vast amounts of user data. In the mobile health (mHealth) domain, researchers are increasingly discovering the opportunities of log data to assess the usage of their mobile apps. To date, however, the analysis of these data are often limited to descriptive statistics. Using data mining techniques, log data can offer significantly deeper insights.

**Objective:**

The purpose of this study was to assess how Markov Chain and sequence clustering analysis can be used to find meaningful usage patterns of mHealth apps.

**Methods:**

Using the data of a 25-day field trial (n=22) of the *Start2Cycle* app, an app developed to encourage recreational cycling in adults, a transition matrix between the different pages of the app was composed. From this matrix, a Markov Chain was constructed, enabling intuitive user behavior analysis.

**Results:**

Through visual inspection of the transitions, 3 types of app use could be distinguished (route tracking, gamification, and bug reporting). Markov Chain–based sequence clustering was subsequently used to demonstrate how clusters of session types can otherwise be obtained.

**Conclusions:**

Using Markov Chains to assess in-app navigation presents a sound method to evaluate use of mHealth interventions. The insights can be used to evaluate app use and improve user experience.

## Introduction

### Background

The development of wearable technology, particularly in health care, medical, and fitness contexts, provides people with devices that give detailed information about various aspects of their health, for example, by registering users’ daily physical activity in terms of step counts, calories, or by providing detailed information about exercise parameters [1].

At the same time, mobile phone technology is reaching significant adoption and penetration rates in both developed and developing countries [2]. This offers an excellent platform to reach a large audience with health-oriented mobile phone apps. Most mobile phones contain sensors that afford measurement of parameters similar to those measured by wearables, including accelerometer and global positioning system.

Mobile health (mHealth) apps are increasingly available to people worldwide. Over 318,000 mHealth apps are estimated to be available in app stores around the world [3]. These apps target various conditions and behaviors including nutrition, smoking cessation, mental conditions, diabetes, and physical activity [4-6]. However, although mHealth apps can empower people to take their health more into their own hands [7,8], research indicates that the uptake of these apps does not necessarily result in sustained usage [9,10]. In digital behavior change interventions (DBCIs), which use mHealth apps to promote health behavior, this lack of engagement with the technology and intervention too often results in high levels of dropout [11,12].

Engagement with DBCIs can be considered from different perspectives. Perski et al [12] define engagement both in terms of a subjective experience and a behavior. The first considers engagement as the user’s subjective experience with the technology, with quality of experience, immersion, usability, and enjoyment as the main determinants of active and frequent usage, whereas the latter takes more objective parameters into account such as frequency and duration of use or use of specific content of the intervention. Yardley et al [11] distinguish between micro and macro levels of engagement, with microengagement referring to specific interaction with the technology and macro levels of engagement considering the engagement with technology within the overall aim of the DBCI to encourage behavior change. This study focuses specifically on this micro level of the engagement with the *Start2Cycle* app, developed to promote recreational cycling. Therefore, it was decided to only analyze the log data of the app and not take any cycling outcome data of the participants into account. Furthermore, as the field trial in which the app was tested was a usability test rather than a full randomized controlled trial, using the outcome data could be misleading in terms of effectiveness.

Measuring engagement with DBCIs receives ample attention. To date however, no consistent or standardized methodology to quantify and measure participant engagement in DBCIs is available [11]. In addition to self-report-based questionnaires distributed after usage of the app, the analysis of log data (anonymous records of real-time actions performed by each user) has been suggested as an opportunity to study actual usage of apps both in the development phase and when the final version has been launched [13] and to tailor interventions [14]. In human-computer interaction research, data mining and machine learning techniques are frequently used for various purposes such as finding user activity patterns in apps and games [15-17], user segmentation [18], and app development [19]. In mHealth research, however, analysis of log data remains often limited to descriptive analysis in terms of number of visits, length of visits, and page views. Although these statistics offer an initial insight into *when* and *how often* participants use mHealth apps, they do not provide us with more profound insights on *how* participants are using them. This information is essential to the development of mHealth interventions and the attainment of their anticipated health effects. Limited research applies more detailed analysis of log data of electronic health apps. Arden-Close [20], for example, used sequence clustering to develop a visualization tool to monitor the use of a Web-based weight management intervention. Furthermore, Miller et al [21] present a framework for analyzing engagement data of health interventions, including suggestions for analyzing log data.

### Objectives

This study partly builds on these insights by adding Markov Chain analysis to the toolkit for analyzing log data of mHealth interventions. In multimedia research contexts, Markov Chains have been frequently used to study Web page navigation [22-24]. In these analyses, Markov Chains are used to model a *trail*, that is, an order of Web page views by 1 user within a Web-use session. This study aims to be a methodological example of how this thinking was applied to in-app navigation such that the Markov Chains model the order of page views made by a user within an mHealth app. This provides insight into how the app is used, that is, which types of usage sessions can be identified and if user sessions can be clustered into distinct groups.

## Methods

This section details the content of the *Start2Cycle* app, the setup of the field trial in which the app was tested, and the methods applied to the app log data.

### The Start2Cycle App

The *Start2Cycle* app was developed to promote recreational cycling in adult populations. The app was designed for both novice and experienced cyclists. It was developed by the Flemish public broadcaster Vlaamse Radio- en Televisieomroeporganisatie, Ghent University, and Vrije Universiteit Brussel and was available for both Android and iPhone operating system devices.

**Figure 1:**
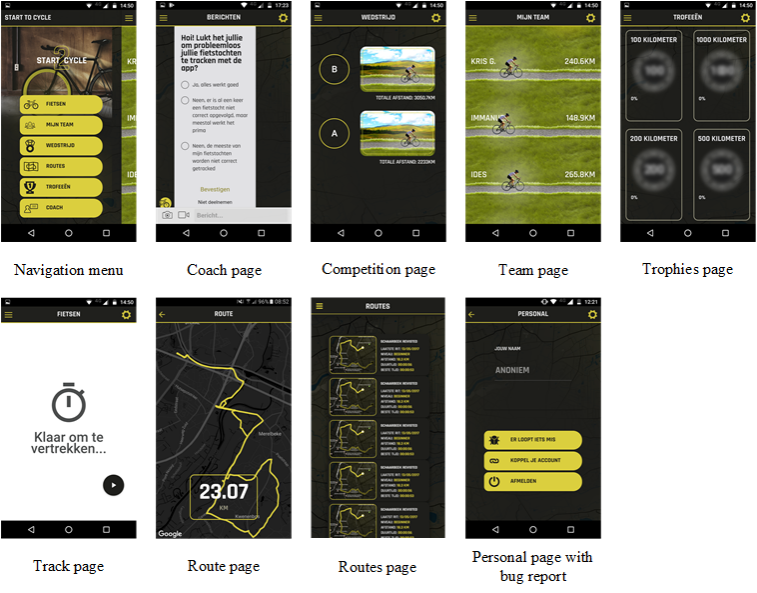
Screenshots of the Start2Cycle app pages.

The app, depicted in Figure 1, has a starting page called the *Coach*. Through this page, 2 researchers provided additional cycling-related content to the app during the field trial by postings polls, news messages, route suggestions, pictures, and movies. Push notifications notified users of new posts. Users could ask questions to the coach. Beyond the starting page, the app had 2 core functionalities: (1) Self-monitoring and feedback on cycling behavior and (2) Rewards and social competition. The latter can be classified as gamification features. The self-monitoring features of the app enable the users to log their cycling activities (*Track* page) and afterwards see the route they took (*Route* page). They were also provided with a page that lists all of the routes they had taken since they started using the app (*Routes* page). Gamification and social features are assumed to have potential to create more engagement with an app and potentially longer lasting behavior change effects [25,26]. Therefore, gamification and social competition and cooperation were included in the app. Users could, for example, collect short videos starring a well-known Flemish sports journalist providing motivational or informational content about cycling on the *Trophies* page. These videos were unlocked at certain *milestones* in the cycling progress of the participant, for example, after his or her first ride and at 25 cycled kilometers. Furthermore, the participants were randomly assigned to 1 of 2 groups (team A and team B), and a competition was set up between the 2 groups. The group with the highest distance cycled (in kilometers) at the end of the trial won the competition. The participants could monitor the performance of their group in comparison to the other on the *Competition* page and the individual performances within their group on the *Team* page of the app. By combining both cooperation among group members and competition between the groups, it was aimed to make the app appealing to both competitive and noncompetitive participants [27,28].

Finally, there was a *Personal* page on which the participants could change their settings, enter or change their screen name, and report errors.

### Field Trial

A 25-day field trial of a prototype version of the *Start2Cycle* app, with primarily a usability testing purpose, was set up with 22 participants in Flanders, Belgium in September 2017. The participants were recruited from a group of people that registered for a 1-week cycling holiday that started at the end of the field trial. No inclusion criteria were set. Anyone joining the cycling holiday could participate in the trial. Participants were asked to log every cycling activity during the 25 days preceding the cycling holiday. The project was approved by the institutional review board, and informed consent was obtained from the participants.

Every action the participants performed in the *Start2Cycle* app was logged. More specifically, per user, each action (or *click*) was logged, along with the time stamp. No session identifier (ID) was collected. Consequently, sessions were identified by grouping actions of a user together, between which no longer than 30 min of inactivity occurred. Using this heuristic, 824 sessions were identified. At the end of the field trial, an online questionnaire was sent to the users to evaluate the use of the app in terms of perceived usefulness, ease of use, motivational potential, enjoyment, and informativeness. The evaluation was conducted using 7-point Likert scales ranging from 1=*Totally disagree* to 7=*Totally agree*.

### Markov Chain Analysis

The log data of the *Start2Cycle* app were analyzed by means of Markov Chain analysis. Markov Chains are a sound method to model stochastic processes and have already been successfully adopted in a wide spectrum of applications ranging from auto-completion while typing [25] to the world-renowned ranking algorithm of the Google search engine [29]. They are defined by a state space *S*, a transition matrix *P*, and optionally an initial state distribution *π*. In the case of this study, we have a discrete state space, defined by the *n* different pages in the mobile app (*S*={s_1_,..., s_n_}). Moreover, we assume the Markov Chain to be of order *1*. In other words, we assume that the probability to transition to a next state depends only on the current state and not on the history of previous states.

To create a Markov Chain, a transition matrix *P*, in which each element on row *i* and column *j* (*p*_i,j_) indicates the probability that a user moves from page *s*_i_ to page *s*_j_, must be constructed. This transition matrix can be constructed from raw data logs containing a *time stamp*, *session identifier*, and the *performed clicks (source* and *destination)*. The session identifier is optional and can be calculated using a heuristic based on the *user identifier* and time stamp of each data log. The number of transitions s_i_ → s_j_ can then be counted to form a matrix *M*. Afterwards, the values in *M* are normalized such that each row sums to 1 to result in *P*.

Furthermore, a surrogate exit-state *s*_n+1_ to which a user transitions when he leaves the app (*exit* page) was created. The initial state distribution *π* can be estimated by counting the first visited state of every session.

### Sequence Clustering

When the number of pages in an app becomes large, manual interpretation of the visualized Markov Chain can become cumbersome. An advantage of having a Markov Chain as underlying data structure of the visualization is that various automated analyses are possible. These can be used to automatically extract insights or to confirm the findings of a researcher. Clustering of different sequences (or trails of user actions) is 1 such analysis [30,31]. To do this, an expectation-maximization algorithm [32] can be applied, which builds a representative Markov Chain per cluster in an iterative fashion until convergence is reached.

Given a collection of sequences *C*, in which each element is represented by a trail of user actions ({s_1_,..., s_i_,..., s_n+1_}, with s_n+1_ the exit-state), we apply the following steps:

1. Determine the number of clusters: *K*
2. Assign each sequence in *C* to a random cluster *c*_i_ with *i* ∊ *{1,* *...* *, K}*
3. For each *c*_i_, construct a Markov Chain or transition matrix (*P*_i_) based on its assigned sequences.
4. For each sequence in *C*, calculate the likelihood *L(* 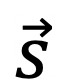, *c_i_)* that the sequence is generated by each Markov Chain by multiplying the probabilities of the different transitions (eg, L({s_0_, s_3_, s_1_, s_n+1_}, c=*p*_0,3_ * p_3,1_ * p_1,n+1_). Then assign the sequence to the cluster with the maximal likelihood: *argmax*_i_*(L(* 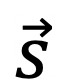, *c*_i_*))*.
5. Reconstruct each Markov Chain, based on the assigned sequences and repeat steps 4 and 5 until convergence. To check convergence, the assignment of sequences to clusters can be compared with the assignment of the previous iteration.

The output of this algorithm is a mapping from each sequence to a cluster *c*_i_ and *K* different Markov Chains that represent each cluster. These Markov Chains can be used to classify new unseen sequences of actions.

### Web-Based Visualization Tool

To ensure a more intuitive interpretation of such results for researchers, interactive visualizations of the results can be created. For this study, such an interactive visualization was developed using D3.js [33], a javascript library, and can thus be displayed in a Web browser. The tool offers both static and interactive visualization to the researcher, which afford adaptation of the visualization in terms of (1) a slider that defines a threshold for a minimum value the corresponding probability of an edge must have to be displayed, (2) enabling and disabling certain pages or nodes and corresponding edges from the visualization, and (3) the ability to drag nodes around to create a nice structured topology. Moreover, simulations of user sessions, based on the provided data, can be performed, and the results of the sequence clustering can be inspected.

## Results

This section details the results of the analysis. First, an overview of the sample is given followed by the results of the Markov Chain and sequence clustering analyses.

### Descriptive Statistics

The app was tested by 22 participants, 18 of which (82%) were male and 12 of which (55%) had a higher education. The evaluation survey, distributed at the end of the field trial, was completed by 16 users (73%). The mean age was 50 years (SD 11.17). Survey results indicated that the app was positively evaluated by the participants. It was considered to be fairly useful (mean 4.63, SD 1.15), easy to use (mean 4.50, SD 1.46), and fun (mean 4.38, SD 1.31). Users rated the app as somewhat motivating (mean 4.25, SD 1.39). The app was scored lower in terms of informativeness (mean 4.06, SD 1.18).

**Table 1:** Start2Cycle app page views.

Page	Views (absolute), n	Views (relative), %
Coach	1159	26.9
Competition	578	13.4
Route	547	12.7
Team	539	12.5
Routes	529	12.3
Track	507	11.8
Personal	268	6.2
Trophies	119	2.8
Bug report	58	1.3
Total	4304	100

After data cleaning, the log data showed that the participants averaged 37 app use sessions (SD 29.35), with an average of 5 actions (SD 5.86) per session during the field trial. The high SDs indicate substantial variability in activity logging between the participants.

Table 1 depicts the number of views per app page during the field trial. It is clear that the *Coach* page was visited the most, although it has to be noted that this was the default starting page for a session. The table further demonstrates that the gamification features on both the *Competition* and *Team* page were frequently viewed, as well as the route tracking features (*Track*, *Route*, and *Routes* pages). This is straightforward, as without users tracking routes, no gamification could be enabled. The *Personal* and *Trophies* page were not often frequented by the users. The same applies for the *Bug report* page.

### Markov Chain Analysis

**Figure 2:**
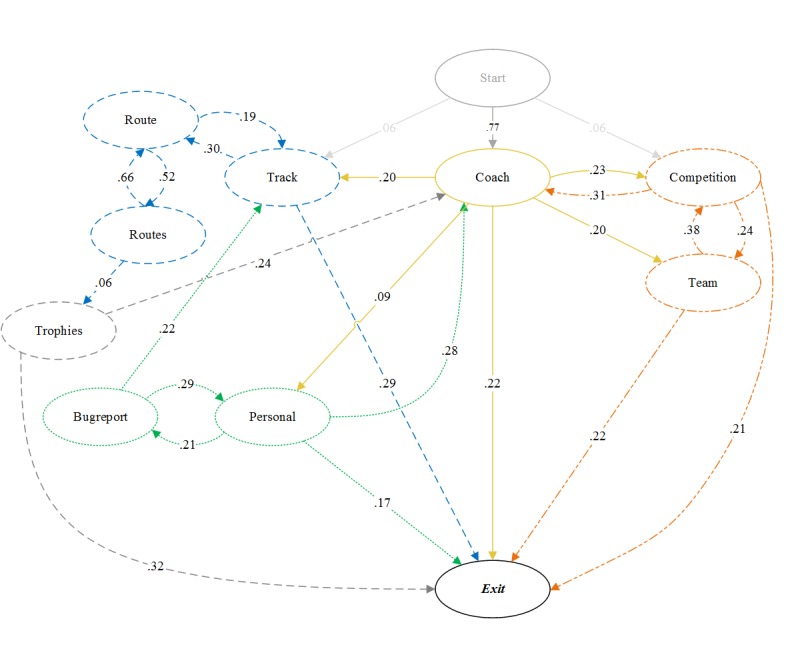
Start2Cycle navigation visualization.

Table 2 demonstrates the transition matrix of the Markov Chain, and Figure 2 visualizes the transitions between the app pages. Not all transitions are displayed in Figure 2. A cut-off of a 0.13 probability was used to improve readability. For the *Trophies* and *Personal* page, the highest incoming probability is also displayed to improve readability and logic of the visualization. Without these incoming probabilities, the pages would be visually isolated in the figure as all incoming edges have a probability lower than 0.13.

Figure 2 demonstrates that the majority of the sessions (76.9%, 634/824) start from the *Coach* page, which was intended by design. The *Coach* page, thus, served as the point from which most of the users started actions from in the app (yellow lines in Figure 2) and returned to before exiting the app. The remaining 23.1% (190/824) of sessions start from other pages than the *Coach* page. This is because of users not exiting the app (eg, switching to another app) and then reopening the app on the same page they left off, most often the *Track* and *Competition* page.

**Table 2:** Transition matrix (probability of transitioning from page A [row] to B [column]). Rows sum up to 1.

	Trophies	Bug report	Coach	Competition	Personal	Route	Routes	Team	Track	Exit
Trophies	—^a^	—	0.24	0.11	0.04	—	0.08	0.12	0.08	0.32
Bug report	—	—	0.12	0.09	0.29	—	0.05	0.12	0.22	0.10
Coach	0.03	—	—	0.23	0.09	0.00	0.04	0.20	0.20	0.22
Competition	0.05	—	0.31	—	0.05	0.01	0.09	0.24	0.05	0.21
Personal	—	0.21	0.28	0.04	—	0.00	0.08	0.13	0.09	0.17
Route	—	—	0.13	—	0.04	—	0.52	0.01	0.19	0.11
Routes	0.06	—	0.07	0.03	0.03	0.66	—	0.05	0.04	0.07
Team	0.04	—	0.11	0.38	0.08	0.00	0.11	—	0.06	0.22
Track	0.01	—	0.13	0.03	0.05	0.30	0.07	0.12	—	0.29
Exit	—	—	—	—	—	—	—	—	—	—

^a^No transitions occurred between the two corresponding app pages.

Visual inspection of the transitions between the different app pages roughly exposes 3 *Start2Cycle* app session types. A first session type can be labeled a *gamification* session (orange striped lines). After launching the app, users would then go from the *Coach* page to either the *Competition* page, to see whether or not their team is still ahead of the other in terms of cycled kilometers (23%) or check how they are ranked within their own team (on the *Team* page, 20%). Switching between these 2 pages is a likely next action. These sessions are often followed by a return to the *Coach* page, particularly from the *Competition* page (31%), or the app session is ended.

Second, a *route tracking* session can be distinguished (blue striped lines in Figure 2). In such a session, a user would start the app with the intention of logging a cycling activity. There is a 20% probability that a user would start the app with this action in mind from the *Coach* page. After tracking a route, there is a high probability that the user will check the route they rode (on the *Route* page) or other routes they have previously cycled (on the *Routes* page) and switch back and forth between these 2 pages. Eventually, the user is likely return to the *Coach* page (13%) and leave his session or perform new actions from there. The app is often left from the *Track* page (29%). This is likely because of the fact that an inactivity of longer than 30 min occurred because of cycling or because of errors in route tracking in the early phases of the trial (a known problem of the app), which prompted the user to leave the app. As could be derived from the limited number of page views displayed in Table 1, the *Trophies* page (grey striped lines), where a user could check whether he or she unlocked a new video in the app, has very limited incoming transitions, the highest being an incoming transition from the *Routes* page. After checking the *Trophies* page, there is a high probability the user will either leave the app (32%) or proceed to the *Coach* page (24%).

The last session type is labeled a *bug report* session (green dotted lines). These sessions were not as frequent as there is only a 9% probability that a user would go from the *Coach* page to his personal page and subsequently report an error or malfunction in the app. When reporting these tracking errors, users often immediately switched to the *Track* page, most likely for a new attempt to track their activity.

### Sequence Clustering

**Figure 3:**
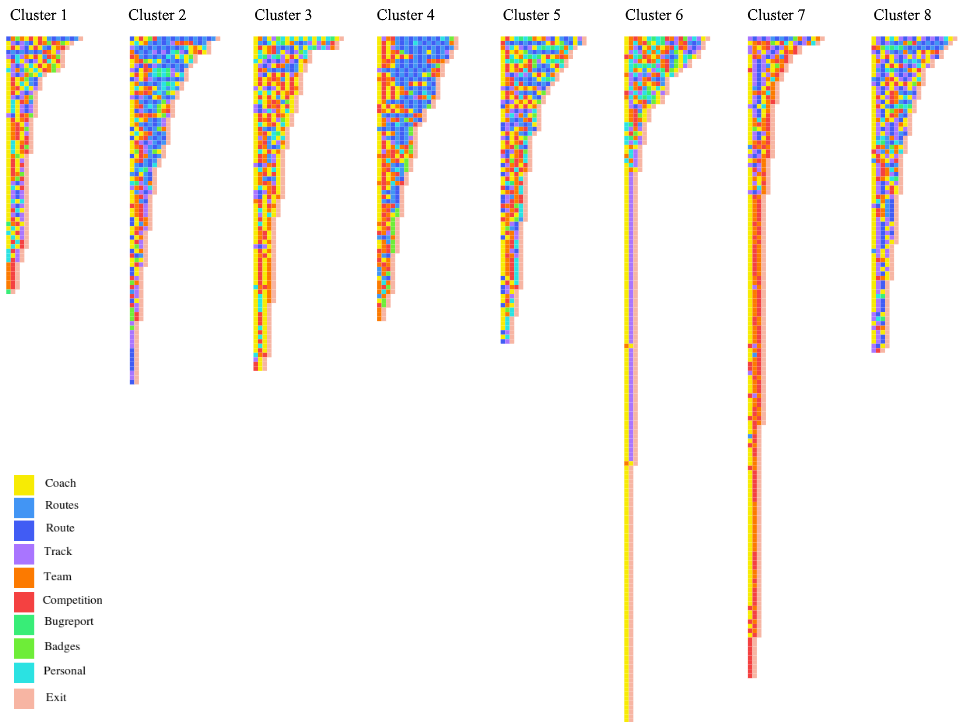
Visualization of sequence clustering.

Although 3 types of user sessions were identified through visual inspection, the actual sequences in the data are more complex. As an example, a session could be considered a *route tracking* session when use of the *Track* page and consequent use of, for example, the *Route* and *Routes* page occurs, but in reality, a user is likely to use gamification features or the coach multiple times in the same sessions. Nevertheless, applying the sequence clustering algorithm with varying *K* clusters, distinct clusters containing specific session types, could be observed. The results of the algorithm are an assignment of each sequence (or user’s session) to 1 of *K* clusters and first-order Markov Chains, 1 for each cluster. Figure 3 displays the assignment of the sessions in *K*=8 clusters.

Only sequences with a length between 2 and 20 actions are depicted in the figure to avoid cluttering. Each row in the visualization in Figure 3 corresponds to a session, and each cell in the row corresponds to a certain action made by the user. Although there is an overlap between clusters, particularly for longer sessions with a varying number of different actions, the tails of the clusters consist of shorter sessions with clear purposes. Cluster 7 shows clear abstraction of gamification sessions. These sessions are typically short and consist of a user opening the app, checking the leaderboards, and exiting the app. Furthermore, cluster 6 makes a distinct grouping of presumed erroneous route tracking sessions consisting of the sequence *Coach*-*Track*-*Exit*. Clusters 2 and 4, on the other hand, distinctively contain more sequences involving checking of the *Route* and *Routes* pages.

**Figure 4:**
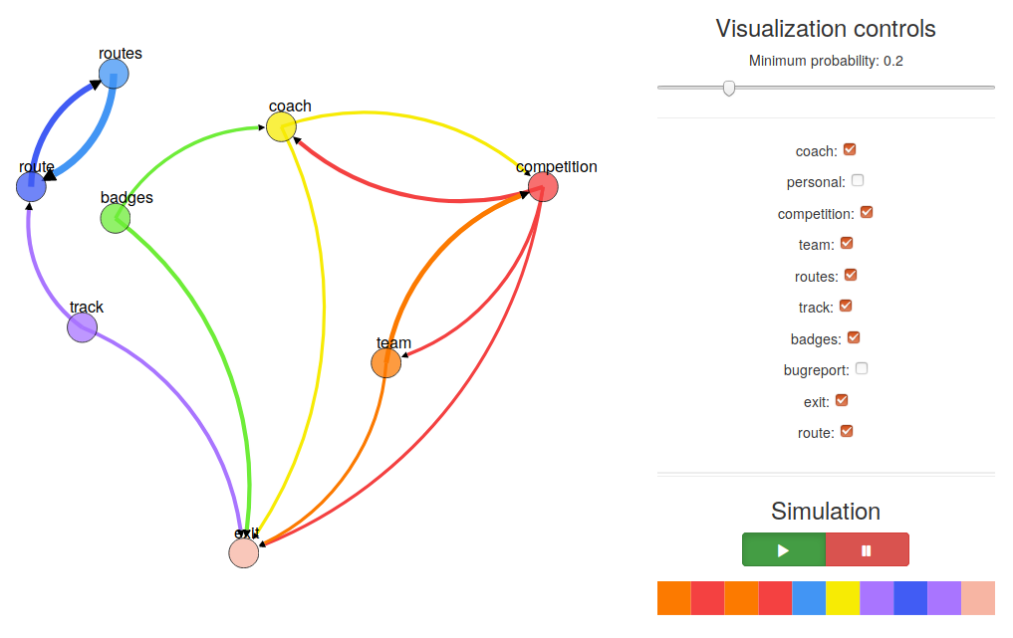
Screenshot of the visualization tool.

### Web-Based Visualization Tool

Figure 4 presents a screenshot of the visualization and simulation tool that was developed to allow researchers to dynamically explore the *Start2Cycle* data. In the tool, researchers can drag around selected nodes, explore transitions from given probability cut-offs, and run simulated paths through the *Start2Cycle* app.

## Discussion

### Principal Findings

The purpose of this study was to demonstrate how data mining can be applied to evaluate the development of mHealth apps. It is clear that by performing a Markov Chain analysis on the log data of the *Start2Cycle* app, it was possible to identify different types of user sessions, to assess the popular features of the app, and cluster frequently occurring app usage patterns.

In the light of further development of the app, this type of analysis has provided insights that may not have been discerned using traditional methods such as usability tests, evaluation surveys, or expert reviews, particularly when dealing with apps with a high number of app pages. Briefly stated, using Markov Chains affords mHealth developers and researchers to assess whether the use of, and the navigation in their app, happens as they envisioned it. In our analysis, 2 distinct types of app usage sessions could be identified, which aligned with the envisioned use of the app: a *gamification* session and a *route tracking* session. Evidently, the *Start2Cycle* app is not excessively complex, so use of the app could be expected to be straightforward. However, this was not entirely the case.

It was clear that the gamification features involving competition and cooperation within and between teams included in the app were appealing to the users, which was expected based on the review of literature on gamification [34,35]. Users often checked these gamification pages and navigated smoothly from one to another, which could be derived from the transition probabilities calculated in the Markov Chain analysis. On the other hand, the trophies concept was clearly less appreciated despite being easily accessible through the navigation menu. The Markov Chain analysis showed little incoming navigation to the *Trophies* page. It appears that the concept behind this feature should be improved to make it more appealing. Anecdotal conversations with some of the respondents revealed that the video content did not appeal to the users, and more tangible trophies (eg, coupons, water bottles, or cycling jerseys) or virtual badges were preferred. Previous research on the effectiveness of badges has demonstrated mixed results [36]. Further research is required to assess the effectiveness in the particular context of this study.

Furthermore, Markov Chain–based sequence clustering demonstrated how similar sequences of actions can be grouped together in clusters. This method becomes particularly interesting when apps contain significantly more pages than the *Start2Cycle* app used in this study, and interpretation from matrices and visualizations is less straightforward. Furthermore, by using this technique, a participant’s app use profile can automatically be determined based on the types of sessions performed throughout the use of the app. The user interface of the app, for example, could then be tailored specifically to this profile.

The application of Markov Chains is only 1 method within a broader range of methods in the context of data mining. The vast amounts of data generated by mHealth apps and games are still largely unexplored. This study aimed to illustrate the potential of 1 method to generate useful insights from log data that can foster further and improved development of mHealth apps and games. Data mining can advance behavior change research by uncovering usage patterns and insights that could not be uncovered through traditional methods.

### Limitations

Some limitations of this study should be noted. The aim was to demonstrate how data mining, Markov Chain analysis in particular, can be applied in the development of mHealth apps. The data used in this study originate from a field trial with primarily usability testing purposes. Therefore, certain properties of the sample may influence how the app was used. For example, although the app is designed to motivate adults to start cycling or to cycle more often, some of the participants in this study were already highly involved in active cycling. It was opted to work with active cyclists to ensure that sufficient (activity) log data would be generated during the field trial. This implies that the sample used in this study is presumably not representative of a population that would typically be asked to use the app. However, as the purpose of this study is primarily to demonstrate the potential of Markov Chain analysis in the development of mHealth apps, the characteristics of the sample can be considered somewhat less important.

Second, it was chosen to use first-order Markov Chains, which assume that the probability of moving to a next state depends only on the current state (and thus not on previous states). This benefits the intuitiveness and simplicity of our analysis. A first-order Markov Chain does not change, although the representation of a higher-order Markov Chain would have to be conditioned on the previous transitions. Clearly, this would make the proposed visualization much less comprehensible. Furthermore, a substantial amount of data is needed to construct a Markov Chain of order *m*>1 that is a good representation of the global user behavior. If the chosen *m* is too high, given the number of samples, then most history state sequences of length *m* do not occur in the data, or only occur infrequently, which causes the transition matrix, conditioned on that history sequence, to be constructed from insufficient data.

Finally, the user sessions used in the analyses were obtained using a heuristic based on time between actions in the app. Better would have been to collect a session ID along with every user session. This was overlooked in the design of the app and the study. Therefore, cut-offs between session may not be entirely accurate. It is recommended for future studies that session IDs are collected along with the data.

### Future Research

Further research should build on the insights of this study and explore how data mining can help to improve mHealth behavior change research. Other data mining techniques are available, and studies using machine learning algorithms, that is, predictive modeling, are strongly encouraged as these could help predicting user actions in the app using different input variables such as user profile variables or placement of different elements on a page. If an accurate model of app usage can be constructed, then the parameters (such as the placement of an element) can be manipulated to maximize the probability that a user takes a certain action. Furthermore, identified usage patterns could also help predicting user attrition and consequently allow an app to take appropriate actions. For example, detecting that a user is likely to drop out of an intervention by his usage pattern can trigger an app to engage the user again at an appropriate moment. However, such analyses would require a larger number of participants to be monitored for an extensive amount of time to detect whether or not they drop out. Moreover, user attrition does not necessarily imply that the participant failed to adopt a healthier lifestyle. It may well be that he or she has no need for an app to support behavior change any longer. In this study, data from 22 participants was used and was sufficient for obtaining insightful results. We recommend higher sample sizes, however.

It should be noted that evidently there is a limit to what can be learned through automated analyses. Additional use of traditional quantitative and qualitative techniques such as surveys and interviews will yield a more complete picture of how the user evaluates the app. In this study, routes were often not tracked at all, the tracking screen froze, all data were lost, or the tracking was fine but the kilometers were not added to the total team score, which could be frustrating to competitive users. Reports of such bugs are best detected using evaluation surveys. Furthermore, correct data selection and cleaning can be a time-consuming and difficult undertaking, and a health researcher may not always be trained to do this.

### Conclusions

This study demonstrated the potential of data mining in development and evaluation of mHealth apps. Results demonstrate how health researchers and developers in the field of mHealth can use and learn from new data analysis methods originating from the field of data mining. Our study shows how usage patterns of mHealth apps can be revealed and provide new insights to how interventions are used. As such, these insights can lead to further improvement of mHealth interventions and consequently improve their effectiveness.

A plea for interdisciplinary research and collaboration with data scientists is therefore required. In this regard, development of visualization and simulation tools that make data mining methods and results more accessible to researchers and developers with limited data mining skills is highly encouraged.

### Availability of Data and Materials

All experiments and calculations in this study were performed using Python 3. All data and code to reproduce the experiments performed in this study, or to create the proposed visualization based on your app logs, can be found on our GitHub page [37,38] under an open license for noncommercial use.

## Abbreviations

DBCIs: digital behavior change interventions
ID: identifier
mHealth: mobile health
